# Successful application of the innovation process to a case of Floyd Type I tracheal agenesis

**DOI:** 10.1016/j.sopen.2022.11.005

**Published:** 2022-12-05

**Authors:** Alicia Greene, Yiqi Zhang, Onur Asan, Joseph B. Clark, Barry Fell, Kevin Harter, Thomas Samson, Dino Ravnic, Robert E. Cilley, Peter Dillon, Donald Mackay, Anthony Y. Tsai

**Affiliations:** a Division of General Surgery, Penn State Children's Hospital, Hershey, PA, USA; b Department of Industrial and Manufacturing Engineering, Penn State University, University Park, PA, USA; c School of Systems and Enterprises, Stevens Institute of Technology, Hoboken, NJ, USA; d Division of Pediatric Cardiac Surgery, Penn State Children's Hospital, Hershey, PA, USA; e Center for Medical Innovation, Penn State College of Medicine, Hershey, PA, USA; f Division of Plastic Surgery, Penn State Children's Hospital, Hershey, PA, USA; g Division of Pediatric Surgery, Penn State Children's Hospital, Hershey, PA, USA

**Keywords:** Innovation process, Tracheal agenesis, Surgical innovation, Needs finding, Pediatric surgery

## Abstract

**Background:**

Innovation is broadly defined as the act of introducing a new product, idea, or process. The field of surgery is built upon innovation, revolutionizing technology, science, and tools to improve patient care. While most innovative solutions are aimed at problems with a significant patient population, the process can also be used on orphan pathologies without obvious solutions. We present a case of tracheal agenesis, a rare congenital anomaly with an overwhelming mortality and few good treatment options, that benefited from the innovation process and achieved survival with no ventilator dependence at three years of age.

**Methods:**

Utilizing the framework of the innovation process akin to the Stanford Biodesign Program, 1) the parameters of the clinical problem were *identified*, 2) previous solutions and existing technologies were analyzed, newly *invented* solutions were brainstormed, and value analysis of the possible solutions were carried out using crowd wisdom, and 3) the selected solution was prototyped and tested using 3D modeling, iterative testing on 3D prints of actual-sized patient parts, and eventual *implementation* in the patient after regulatory clearance.

**Results:**

A 3D-printed external bioresorbable splint was chosen as the solution. Our patient underwent airway reconstruction with “trachealization of the esophagus”: esophageotracheal fistula resection, esophagotracheoplasty, and placement of a 3D-printed polycaprolactone (PCL) stent for external esophageal airway support at five months of age.

**Conclusions:**

The innovation process provided our team with the guidance and imperative steps necessary to develop an innovative device for the successful management of an infant survivor with Floyd Type I tracheal agenesis.

**Article summary:**

We present a case of tracheal agenesis, a rare congenital anomaly with an overwhelming mortality and few good treatment options, that benefited from the innovation process and achieved survival with no ventilator dependence at three years of age.

The importance of this report is to reveal how the innovation process, which is typically used for problems with significant patient population, can also be used on orphan pathologies without obvious solutions.

## Introduction

Innovation is broadly defined as the act of introducing a new product, idea, or process which requires invention and implementation [1]. This entails an iterative process that helps teams identify unmet clinical needs, invent solutions, and implement ideas. The field of surgery is built upon continuous innovation, revolutionizing technology, science, and tools to improve patient care [2]. Medical device innovation is a significant factor in the practice of health care and is the particular focus of the Stanford Biodesign Fellowship Program, which utilizes the innovation process.

The innovation process should be based on a participatory design approach where the end users are involved in the process from the beginning to the end and iterations can be taken at any point to refine the solution. This process starts with identifying a need, termed needs-finding phase. A need statement is then developed, which encapsulates the clinical problem that has been identified, the specific population afflicted by the problem, and a measurable outcome that can be affected by potential solutions [3]. If there are multiple needs then a screening process takes place to identify the main need. The screening process has filters that include the clinical impact of the need, the degree of understanding of the pathophysiology involved, a consideration of the existing and emerging clinical approaches, and a preliminary assessment of the market potential for a solution to this need, although in this case, availability of other solutions and not marketability of the proposed solution was the focus [3].

Once there is a clear needs statement identified, the team can begin the inventing, or design, phase. Brainstorming sessions are conducted where several potential solutions are discussed. The team must refine the list of solutions, filtering by considerations such as regulatory pathways, reimbursement potential, technical feasibility, and viability of the model needed to bring the solution to the patients [3]. Research will often have to take place to gain knowledge on these considerations for each potential solution. Development of alternative designs for comparison is then generated. The team then begins developing and prototyping the top solutions, often times requiring multiple iterations of prototypes for each concept [3]. The final concept is then selected.

The final phase in the innovation process is implementation. Many medical devices or solutions will require clinical, regulatory, reimbursement, marketing, and sales strategy planning. Funding sources may need to be investigated and lastly, licensing or regulatory clearance will need to be obtained.

We present a case of tracheal agenesis, a rare congenital anomaly with an overwhelming mortality and few good treatment options, that benefited from the innovation process and achieved survival with no ventilator dependence at three years of age.

## Methods

Utilizing the framework of the Innovation Process akin to the Stanford Biodesign Program, the parameters of the clinical problem were identified and previous solutions were analyzed. Existing applicable technologies and newly invented solutions were collected and compared, and value analysis of the possible solutions were carried out using wisdom of the crowd, i.e., collective opinion of a group of individuals rather than that of a single expert. This was done by providing a panel of experts from various clinical disciplines, engineers with innovation experience, and business leaders with a detailed description of the solutions with their pros and cons and polling their vote in an email survey. This panel of experts was selected based on their experience in the process of innovation or entrepreneurship. In other settings where there may be a plethora of solutions offered, a core team managing the project will use the design criteria for the problem to decide which proposed solutions to eliminate from the list. The top vote-getting solution was then selected, prototyped, and tested using 3D modeling and tested on 3D prints of actual-sized patient parts. The stent was implemented in the patient after regulatory clearance through FDA, Department of Health, IRB, family consent, and hospital approval.

## Results

### Clinical problem identification phase

Our patient was a term, 3.6-kg girl born to a 35-year-old mother with limited prenatal care. During the first week of life the child was diagnosed with tracheal agenesis after bronchoscopy demonstrated a tracheoesophageal orifice in the mid-esophagus communicating to confluent mainstem bronchi. Additional studies also demonstrated a complete atrioventricular canal defect, a right choroid plexus cyst and hemivertebrae.

The parents expressed a strong desire to pursue potential lifesaving measures for their child. The patient was in need of airway reconstruction. The patient initially underwent lower esophageal division and gastrostomy tube placement on day of life (DOL) 3 and duodenojejunostomy and Ladd procedure for duodenal obstruction and malrotation on DOL 8. On DOL 15 the patient underwent upper esophageal division with the upper esophagus diverted to a right cervical esophagostomy (“spit fistula”) and the middle esophagus externalized as a stoma above the sternal notch (“airway esophagostomy”) which was intubated with an extended tracheostomy tube.

A multidisciplinary team was created and parameters of the problem were identified. This team comprised of members from the Penn State Health Department of Pediatric Surgery, Plastic Surgery, Pediatric Cardiothoracic Surgery, Center for Medical Innovation, and Surgical Innovation Group.

### Inventing or design phase

**Fig. 1:**
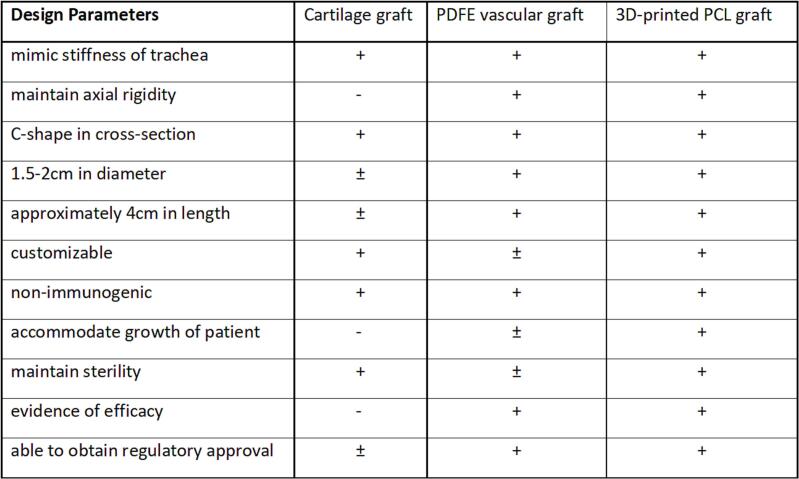
Value benefit analysis of the top three solutions. Cartilage graft takes cartilage from other parts of the patient's body to form the desired c-shaped rings that can be tacked to existing esophageal airway. ePTFE, or polytetrafluoroethylene, grafts are existing ribbed vascular grafts that can be customized to a c-shape cross section. PCL, or polycaprolactone, is a resorbable material that can be 3D-printed and has demonstrated to be biocompatible.

Potential solutions with existing technologies were explored and compared using value benefit analysis (Fig. 1). 3D-printing and pre-operative planning was discussed and agreed upon using crowd wisdom-based decision making. The primary solution selected was a 3-D printed external splint using PCL material that will resorb in 3 years and allow for future growth of the airway.

To test our proposed solution, a life-sized model of the patient's thorax and its contained organs was 3D-printed. Multiple copies of the airway esophagostomy or neotrachea were printed to allow for testing. Variations of the proposed esophagotracheoplasty were also attempted on the 3D models. Different sizes of the 3D-printed PCL splints were checked for fit on the models. The testing demonstrated good fit of the 3D-printed splints over the esophagotracheoplasty.

### Implementation phase

Regulatory clearance was obtained from the US Food and Drug Administration under expanded access, the Pennsylvania Department of Health, Penn State University Institutional Review Board (protocol No. 2018-002SU), family consent, and hospital approval.

At five months of age, the patient was now ready for definitive airway reconstruction with our 3D-printed external bioresorbable splint. She underwent resection of the tracheoesophageal fistula, esophagotracheoplasty and placement of the stent for external esophageal airway support.

The surgery was successful. The patient was discharged home on a home ventilator program at thirteen months of age. She eventually liberated from continuous mechanical ventilation at two and a half years of age and is currently doing well with no ventilator requirement. To note, she has since undergone restoration of gastrointestinal continuity using a colon interposition graft to connect the cervical esophagus to the stomach.

## Discussion

The innovation process focuses on forming interdisciplinary teams to identify unmet clinical needs, invent new solutions, and implement the ideas (the 3 “I's”) [3]. Innovation process can be applied to a variety of clinical areas, including Pediatric Surgery where some rare pathologies may benefit from a novel solution. While most innovative solutions are aimed at problems with a significant patient population, the process can also be used on orphan pathologies without obvious solutions.

**Fig. 2:**
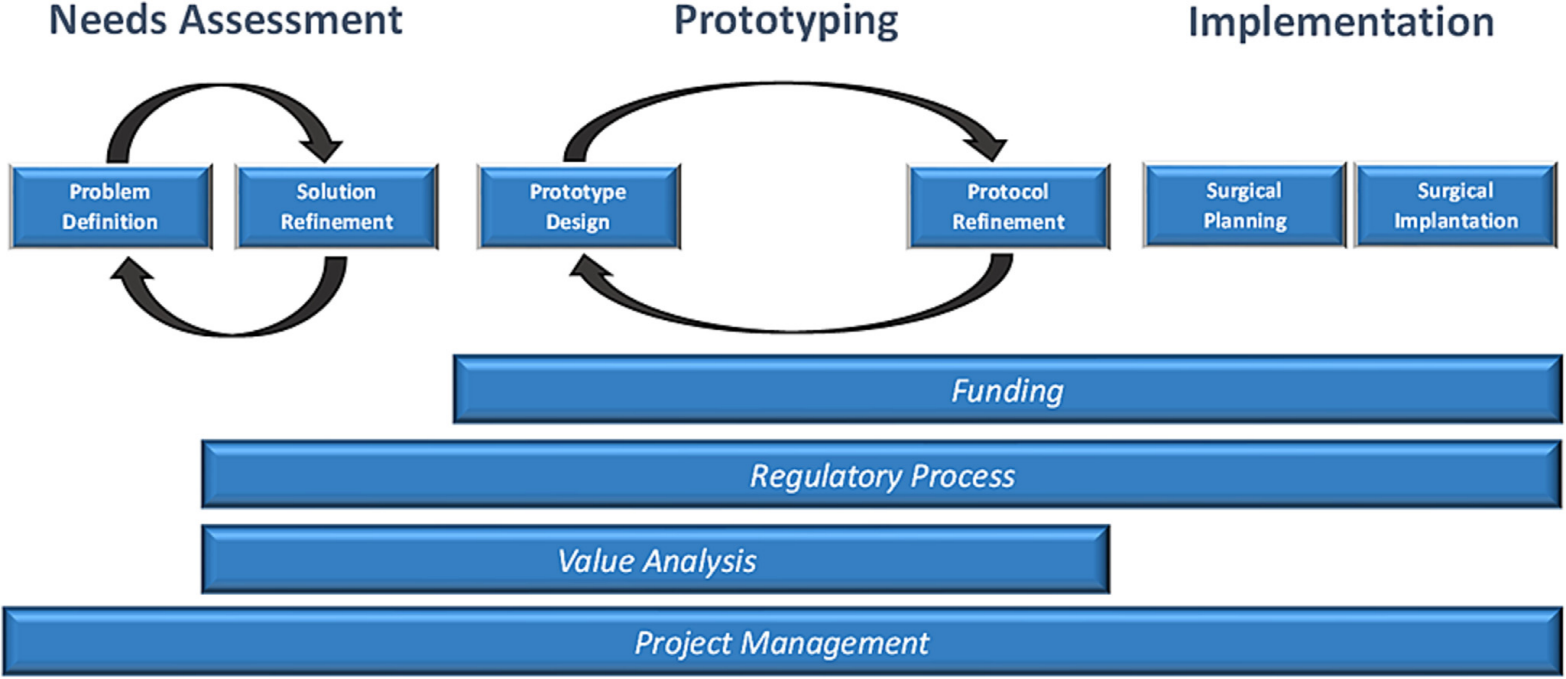
A schematic flow diagram detailing the steps of the innovation process that our team utilized to develop an innovative device for the successful management of an infant survivor with Floyd Type I tracheal agenesis.

Tracheal agenesis is a rare and typically fatal congenital malformation that occurs in less than 1 in 50,000 live births [[4], [5], [6]]. Approximately 150 cases have been reported in the literature with near universal lethality [6]. Tracheal agenesis is typically associated with esophageal communication with the trachea or main bronchi [5]. There have been few successful airway reconstruction approaches described in the literature. The anticipated dismal prognosis was illustrated but the parents expressed a strong desire to pursue potential lifesaving measures for their child. The need in our case was for the creation of a substitute esophageal airway. In our study, the innovation process guided our interdisciplinary team, ensuring there were no critical steps overlooked (Fig. 2).

Prior to generating possible solutions for our needs statement, our team embarked on a screening and research phase. “In the excitement of having identified one or more compelling needs, an innovator's instinct may be to quickly jump ahead and begin inventing. However, establishing a detailed knowledge of the relevant disease state, with a particular focus on its mechanism of action, is fundamental to validating any need and understanding how it can be addressed.^7^” Our team performed an extensive review of the literature on tracheal agenesis, including the anatomy and physiology, pathophysiology, symptoms, outcomes, and epidemiology of the disease.

During this investigative phase, it was discovered that there were very few survivors for this disease with only seven reported cases in Asia and one in the rest of the world [4,5]. All except two of the survivors received external splints. They typically succumb to complications of airway collapse and recurrent airway infections [8,9]. This discovery led to the identified design parameters described in our results section. In addition, it was during this phase that currently available technologies suitable for a potential solution were discovered. Specifically, ePTFE grafts are the most common external splint used in the previous survivors. 3D-printed PCL splints have been used for tracheomalacia and bronchomalacia with good success in pediatric patients including infants [10].

During the design phase, the value benefit analysis clearly identified a preferred solution of the 3D-printed PCL external splint, with the PTFE external graft being a viable backup solution. The use of the wisdom of the crowd confirmed the same result validating our chosen solution. Pursuit of regulatory approval was expeditious given previous precedence. This further reinforces the benefit of a thorough investigative phase where regulatory and all other relevant issues are discovered and considered.

3D-printing was employed not only in our final solution, but also in the refinement of the design of the final solution as the use of the life-sized 3D printed models allowed us to determine the likely dimensions of the required 3D printed splint. CT imaging of the patient was imported into computer-aided design software to create a virtual anatomic model for custom design of the external splint. Prototypes were created and additional benchtop fit-testing was performed with life-size 3D-printed splints and airway models [11]. Multiple design iterations were able to be 3D-printed efficiently without delaying the implementation phase. This iterative prototyping is an essential step through which the innovator learns about functionality, explores features, gathers preliminary feedback, and answers questions that can only be resolved through the manifestation of the design [7].

Tracheal agenesis is a rare anomaly that is associated with overwhelming early mortality and few good treatment options for creating substitute esophageal airways. We demonstrated that using the innovation process, such rare anomalies can benefit from solutions derived from the proven framework of thorough investigation, iterative design prototyping with design parameters derived from the investigation, and final implementation covering regulatory, technical, logistical, financial, and ethical concerns necessary. Similar process has been employed at our institution for the ideation and implementation of solutions to proposed clinical problems under the guidance of our Surgical Innovation Group with successful commercialization in various surgical and medical applications. Similar process was also used for emergency use applications during the COVID-19 pandemic to meet supply chain shortages. At our institution, this is the first application of the process in the pediatric space where orphan pathologies are more likely to be encountered. With such framework, a successful solution is possible even for a dire diagnosis such as tracheal agenesis.

## Funding sources

This research did not receive any specific grant from funding agencies in the public, commercial, or not-for-profit sectors.

## Ethics approval

The stent was implemented in the patient after regulatory clearance through FDA, Department of Health, IRB (protocol No. 2018-002SU approved on March 28, 2018), family consent, and hospital approval.

## CRediT authorship contribution statement

**Alicia Greene:** Formal analysis, Writing – original draft, Writing – review & editing. **Yiqi Zhang:** Validation, Writing – review & editing. **Onur Asan:** Validation, Writing – review & editing. **Joseph B. Clark:** Writing – review & editing. **Barry Fell:** Conceptualization, Investigation, Methodology, Project administration, Writing – review & editing. **Kevin Harter:** Resources. **Thomas Samson:** Methodology, Resources, Writing – review & editing. **Dino Ravnic:** Methodology, Writing – review & editing. **Robert E. Cilley:** Conceptualization, Methodology, Supervision. **Peter Dillon:** Conceptualization, Methodology, Supervision. **Donald Mackay:** Supervision, Writing – review & editing. **Anthony Y. Tsai:** Conceptualization, Data curation, Methodology, Investigation, Formal analysis, Project administration, Supervision, Visualization, Writing – review & editing.

## Declaration of competing interest

None.

There were no financial or personal relationships with other people or organizations that could inappropriately influenced our work.
